# Combined Effect of Pressure and Carbon Dioxide Activation on Porous Structure of Lignite Chars

**DOI:** 10.3390/ma12081326

**Published:** 2019-04-23

**Authors:** Natalia Howaniec

**Affiliations:** Department of Energy Saving and Air Protection, Central Mining Institute, Pl. Gwarkow 1, 40-166 Katowice, Poland; n.howaniec@gig.eu; Tel.: +48-32-259-2219

**Keywords:** lignite, porous structure, carbon dioxide, activation, pressure

## Abstract

Lignite is an important natural resource with the application potential covering present and future energy systems, including conventional power plants and gasification systems. Lignite is also a valuable precursor for the production of porous materials of tailored properties for various environmental applications, including the removal of contaminants from gaseous or liquid media. Although the lignite-based activated carbons are commercially available, various approaches to produce carbon materials of desired properties are still being reported, covering temperature, partial oxidation and chemical activation effects on surface and structural properties of these materials. Limited data is, however, available on the effects of pressure as the activation parameter in shaping the porous structure of carbonaceous materials, in particularly lignite-derived. In the study presented the combined effect of carbon dioxide activation and pressure in the range of 1–3 MPa at the temperature of 800 °C on the development of porous structure of lignite chars was reported. The study was also focused on poor-quality resources valorization by using a relatively low calorific value, low volatiles and high ash content lignite as a carbon material precursor. The results showed that the application of pressure in carbon dioxide-activation process at 800 °C results in generation of chars of comparable or higher specific surface area than the carbon materials previously received with demineralization and carbon dioxide activation of lignite. They also proved that the combined pressure and carbon dioxide activation may be effectively applied in conversion of low quality lignite into valuable porous materials.

## 1. Introduction

Lignite is a considerable element of world coal resources [1]. As such, it is not only an important energy resource applied currently in conventional power plants but also a fuel particularly suitable for gasification and co-gasification systems for its higher reactivity when compared to bituminous coals [2,3,4]. Lignite is also a valuable parent material for the production of porous materials of tailored properties for various industrial applications [5,6,7,8]. The majority of them concerns sorption processes in the removal of contaminants, e.g., phenol, mercury, sulphur oxides, copper, and organic compounds from gaseous or liquid media [9,10,11,12,13,14]. Although the lignite-based activated carbons are commercially available, various approaches to produce carbon materials of desired properties are still being reported. The vast literature is available on the application of the temperature and various oxidizing agents [5,7,11,12,13,14,15,16,17] as well as acidic or basic treatment [7,10,18,19,20] in shaping the surface and structural properties of lignite-derived carbon materials. Limited data is, however, available on the effects of pressure as the activation parameter in shaping the porous structure of carbonaceous materials [21,22,23], in particularly lignite [24,25,26]. A few studies considering the development of porous structure of bituminous coals under carbon dioxide atmosphere [27,28] or inert gas [24,29,30] and elevated pressure are available. Porous structure development of chars is also considered in the literature in terms of lignite suitability and chars reactivity in gasification process, in particularly with the incorporation of carbon dioxide in a valorization cycle as a gasification process reactant [20,31,32,33,34]. Previous studies showed that the values of surface area of bituminous coal chars developed under carbon dioxide atmosphere in general drop with process pressure [27,28]. Swelling properties have been reported to influence the porous structure of coal chars under inert gas atmosphere and pressurized conditions, and although this effect is enhanced with increased coal rank, it also depends on volatiles and specific petrographic components content [24,29] and vary with pressure values [5,30]. The amounts of particular mineral components, differing in rates of expansion, e.g., kaolinite, quartz, pyrite, and calcite affect the porous structure development of lignite chars at increased temperature. The mineral matter in bituminous coal-derived chars was also reported to be severely affected qualitatively and quantitatively when treated with carbon dioxide at 900 °C. The inorganic amorphous phase was decomposed and the reduced mineral forms were oxidized and reacted with aluminosilicates forming calcium and iron minerals [35]. 

The combined effect of carbon dioxide activation and pressure on the development of porous structure of lignite chars has not been reported so far. Therefore, the experimental study on the application of pressure in the range of 1–3 MPa in carbon dioxide-activation of lignite chars was performed and its results are presented in this paper. The lignite of relatively low calorific value and high ash content was selected as a precursor which makes the study valid also in the context of poor quality resources valorization.

## 2. Materials and Methods 

The lignite of relatively low calorific value, high ash and sulfur content, provided by Polish opencast mine from Szczercow deposit, was selected as the chars precursor. Lignite of Szczercow deposit is characterized by high huminite maceral group content, of approx. 82% vol., including densinite share of 44% vol. and atrinite content of 23% vol. The liptinite and intertinite contents are of 7 and 4% vol. Highly porous macerals, like textinite, ulminite and atrinite amount in total to approximately 37% vol. [33]. The mineral matter content is approximately 8% vol. with the dominance of clay minerals. The proximate and ultimate analyses of lignite tested were performed in an accredited laboratory in compliance with the relevant standards and are given in Table 1. 

**Figure 1:**
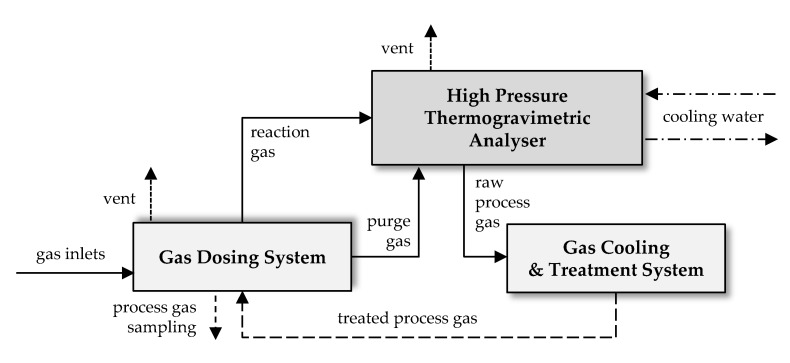
Schematic diagram of the system for carbon materials preparation at high temperature and pressure and with the use of carbon dioxide activation.

Lignite sample of 1 g was heated in a high-pressure thermogravimetric analyzer (Rubotherm GmbH, Bochum, Germany) with the heating rate of 20 °C/min in an argon atmosphere to the final process temperature of 800 °C (see Figure 1) and pressurized to the final process pressure of 1, 2 or 3 MPa, respectively. When the final process temperature and pressure were reached carbon dioxide was introduced to the reactor with a flow rate of 100 mL/min for 120 min. 

The resulting carbon materials were outgassed at 120 °C overnight and analyzed in terms of their porous structure parameters with the application of a gas sorption analyzer Autosorb iQ (Quantachrome Instruments, Boynton Beach, FL, USA). Based on the nitrogen sorption isotherm data acquired at −196 °C, the specific surface area and pore size distribution were determined with the application of the multi-point BET method [36] and the Density Functional Theory (DFT) [37], respectively. The total pore volume was quantified as the volume at the relative pressure of 0.99. The narrow micropore area and volume were further analyzed on the basis of the carbon dioxide isotherm at 0 °C and the Monte Carlo (MC) method [38]. The surface properties of the resulting carbon materials were also explored with the use of a scanning electron microscope SU-3500N (Hitachi High-technologies Corporation, Tokyo, Japan).

## 3. Results and Discussion

**Figure 2:**
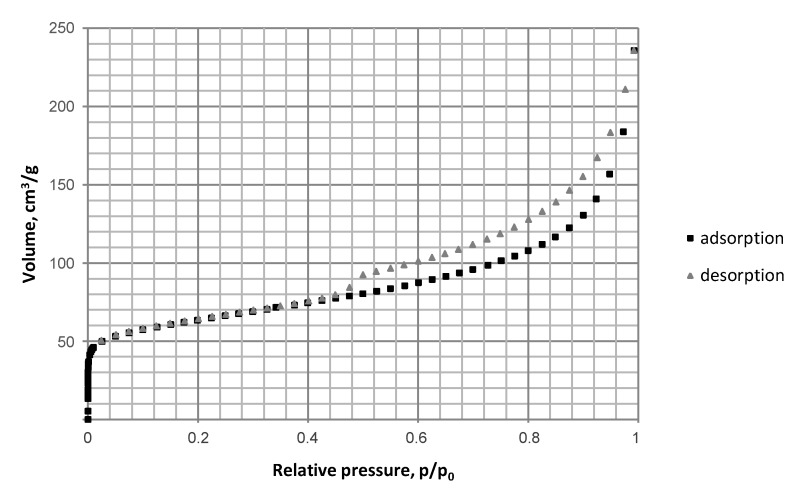
Nitrogen isotherm (−196 °C) for lignite chars produced at 800 °C, under 2 MPa and with carbon dioxide activation.

The porous structure of carbon materials produced from low quality lignite at the temperature of 800 °C and under the pressure of 1–3 MPa with a carbon dioxide activation step was complex and composed of micro- and mesopores. High uptake at low relative pressures, which may be seen in Figure 2, presenting the exemplary nitrogen isotherm for lignite chars tested, is indicative of micropores present in the porous structure of lignite chars. The occurrence of a hysteresis loop proves that the material is also rich in mesopores and its profile reveals the irregular, slit-like shape of pores [39]. 

The results of the porous structure parameters of carbon materials produced under the pressure of 1–3 MPa and at atmospheric pressure, for comparison purposes, are given in Table 2. It may be seen that the rise in pressure resulted in an increased specific surface area values. The highest increase, of 9%, was observed with the change in the process pressure from the atmospheric to 1 MPa. Further increase in pressure in the range 1–3 MPa gave the rise in the specific surface area of 6–7% per 1 MPa. 

The average pore diameter showed a decrease with pressure in the range 0.1–2 MPa (see Table 2). The difference in values of the average pore size of carbon materials produced under 2 and 3 MPa was within the experimental error. It implies that the pressure-enhanced development of smaller pores resulting from the devolatilization, moisture release and partial oxidation of carbon with carbon dioxide under the pressure of up to 2 MPa was counteracted with merging of pores in larger structures under the pressure of 3 MPa. A similar trend was also observed previously for lignite [25,26] and bituminous coal [22,26], as well as biomass chars [21,26] with no carbon dioxide activation, although the limiting values of pressure varied between 2 and 3 MPa for various parent materials. The differences in the values of the total pore volume for chars activated with carbon dioxide under the pressure of 0.1–2 MPa were within the experimental error. However, under the highest pressure tested, of 3 MPa, an increase of approximately 8% in the total pore volume was observed (see Table 2). 

**Figure 3:**
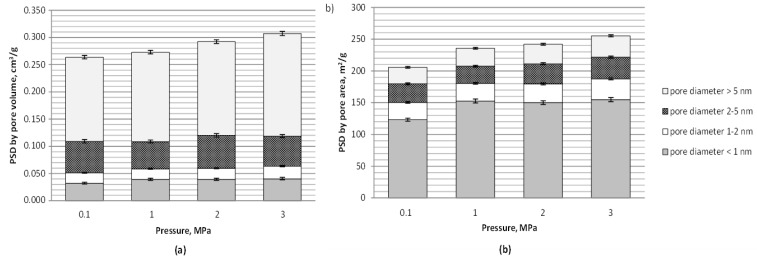
Distribution of: (**a**) pore volume and (**b**) area based on DFT method and nitrogen isotherm (−196 °C) for lignite chars produced at 800 °C, under 0.1–3 MPa and with carbon dioxide activation.

The pore size distribution (PSD) data showed an increase in a pore volume with pressure applied mainly because of increasing volume of mesopores of a diameter over 5 nm in lignite chars with carbonization pressure from 0.1 to 3 MPa (see Figure 3a). The pore volume of small micropores (diameter below 1 nm) determined based on the nitrogen isotherm increased with a change in the pressure from atmospheric to elevated, and was comparable for chars generated under 1–3 MPa. The variation in process pressure seemed to have no measurable effect on the development of 1–2 nm micropore volume with the carbon dioxide activation at 800 °C, which may be related to closure of these micropores or their merging in larger structures at higher pressures. The latter seems to be also demonstrated by the increasing share of mesopores of a diameter over 5 nm. These pores had a dominant role in the increase in the total DFT pore volume with pressure which amounted to 4, 11 and 17% with pressure rise from atmospheric to 1, 2, and 3 MPa, respectively. The dominant share of micropores in shaping the pore area is also visible (see Figure 3b) as well as the positive effect of pressure applied on the development of the smallest pores (of a diameter below 1 nm) and the total DFT area of pores (Figure 3b). Under 3 MPa the share of mesopores of a diameter over 2 nm in the pore area also slightly increased which again may be indicative of merging of pores in larger structures under the highest pressure tested. The total DFT area of pores increased with change of the process pressure from atmospheric to 1, 2 and 3 MPa of 15%, 18% and 24%, respectively. 

**Figure 4:**
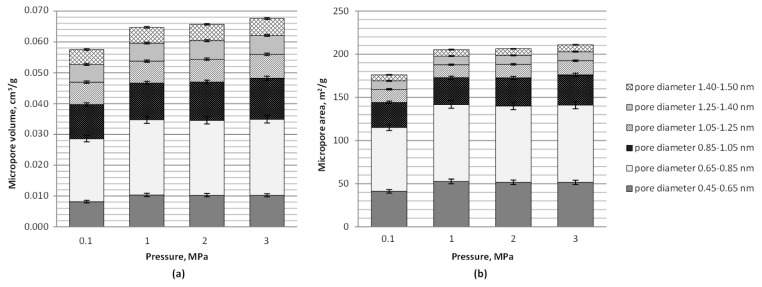
Narrow microporosity determined based on the carbon dioxide isotherm (0 °C) for lignite chars produced at 800 °C, under 0.1–3 MPa and with carbon dioxide activation: (**a**) Micropore volume, and (**b**) micropore area.

The area and volume of narrow micropores (diameter range of 0.45–1.5 nm), determined based on the carbon dioxide sorption isotherm, showed a clear increase with a change in process conditions from atmospheric to pressurized (Table 2). This was caused mainly by an enhanced development of pores of a diameter 0.65–0.85 nm (see Figure 4). No meaningful difference was however observed with further changes in pressure from 1 to 3 MPa in terms of narrow microporosity, except for a slight increase in the volume of pores of a diameter in the range of 0.85–1.05 nm under 3 MPa, giving a slight rise to the total MC pore volume, which may be the effect of structural rearrangements under the maximum pressure tested. 

The activation with carbon dioxide under the increased pressure seems to be resulting in an increased average pore diameter and the total pore volume, as well as a slightly lower specific surface area when compared to values observed previously for lignite-derived materials with pressure, as the only activation agent in carbonization step at 1000 °C [25] or carbon dioxide as the only activation agent at 900 °C [8]. These effects are clearly also related to the temperature and composition of a parent material since in the studies with CO_2_-only activation at 750–900 °C [5] similar values, of 0.336–0.365 cm^3^/g to the reported in this study were observed, but for a lignite of a considerable higher volatiles content (48%) and significantly lower ash content (5%), which made it more suitable as a porous material precursor. This proves that the increased pressure and high temperature may be successfully applied instead of partial CO_2_-oxidation for production of lignite-derived chars of well-developed surface area and relatively low average pore diameter. 

The application of pressure in carbon dioxide activation process at 800 °C enabled production of chars of only slightly lower specific surface area than the ones received with demineralization and carbon dioxide activation step of lignite of a considerably lower ash content (8–9%) than in the study presented here, which implies that pressure may be also considered as an alternative to chemical activation [20]. The total pore volume reported for lignite chars produced with pressure and carbon dioxide activation within the study presented here doubled the values observed for lignite chars of the volatiles content as high as 50% at 800 °C, with chemical demineralization and carbon dioxide activation [11]. 

These results show that the combined pressure and carbon dioxide activation may be effectively applied in utilization of poor quality parent materials (low volatiles, high ash content) for porous materials development. They also prove that the combination of pressure and carbon dioxide activation results in the development of porous structure of lignite-derived materials of a comparable specific surface area and of a similar or higher total pore volume than observed for complex demineralization, chemical activation and carbon dioxide treatment of the respective parent materials. 

**Figure 5:**
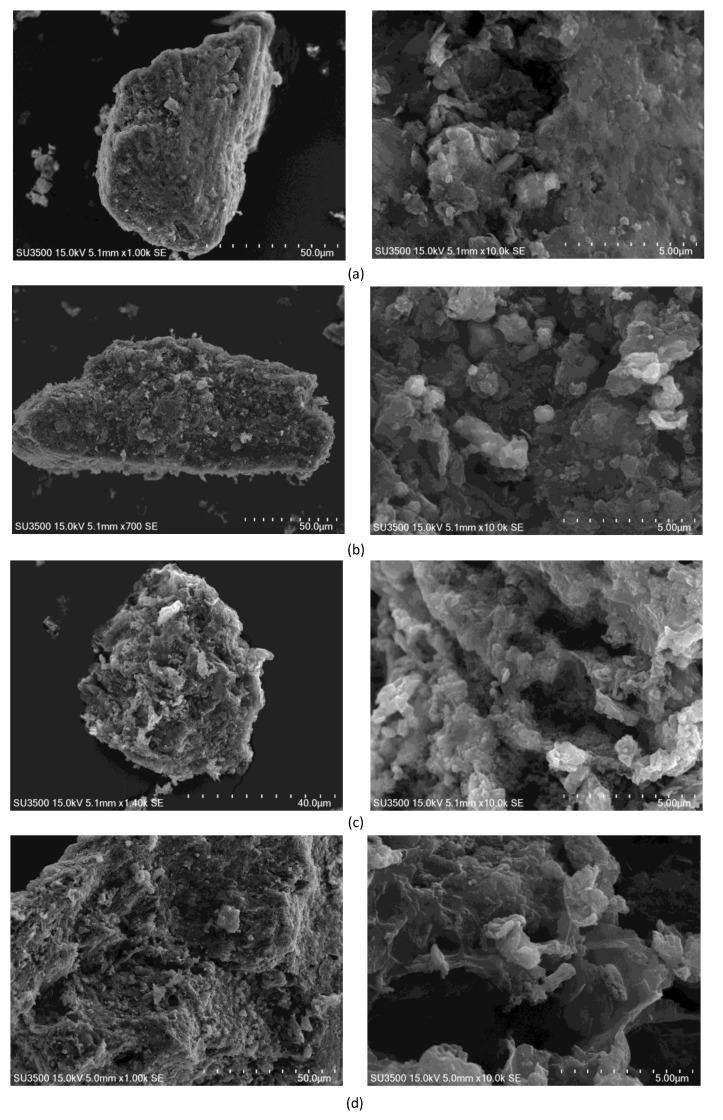
SEM images of lignite chars generated under carbon dioxide atmosphere at 800 °C and under the pressure of: (**a**) 0.1 MPa, (**b**) 1 MPa, (**c**) 2 MPa and (**d**) 3 MPa.

As it can be seen from the SEM (define) images presented in Figure 5, the surface of char particles generated under 0.1 and 1 MPa was visibly smoother (Figure 5a,b) than of chars produced under the elevated pressure of 2 and 3 MPa (Figure 5c,d). However, even for the chars generated under 1 MPa a clear difference may be noticed, consisting in a less dense texture and a more complex structure with some cracks and roughness, when compared to chars developed under atmospheric pressure, resulting from volatiles release and lignite carbonization under carbon dioxide atmosphere. This is in line with the variations in the average pore diameter of chars as described above, as well as increased specific surface area and micropore area with pressure applied (Table 2). The chars developed under 2 and 3 MPa (Figure 5c,d, left) have a more pumice-like structure than a plate-like structure characteristic for chars developed under atmospheric pressure and 1 MPa (Figure 5a,b, left). The chars produced under 2 and 3 MPa (Figure 5c,d, right) showed also visibly more expanded cavities and larger cracks than chars generated under lower pressures. They are likely to be composed of a considerable amount of micropores and smaller mesopores of irregular shape as demonstrated also by the nitrogen isotherms shape and DFT data (Figure 2). This means that the elevated pressure resulted in an enhanced porosity of chars under the experimental conditions applied, though there may also have occurred some rearrangements in the porous structure resulting from thermal annealing observed previously for carbon dioxide treatment of bituminous coal chars at the temperature of 800–900 °C [34]. 

## 4. Conclusions

On the basis of the experimental study performed and presented within the paper the following conclusions may be drawn:

1. The elevated pressure and high temperature may be successfully applied instead of partial carbon dioxide oxidation for production of lignite-derived chars of well-developed surface area and relatively low average pore diameter.
2. Application of pressure in carbon dioxide-activation process at 800 °C results in generation of chars comparable or superior in terms of the specific surface area when compared to carbon materials received with demineralization and carbon dioxide activation of lignite, which implies that pressure may be considered as an alternative to demineralization preceding the carbon dioxide activation.
3. The combined pressure and carbon dioxide activation may be effectively applied in conversion of poor quality lignite into porous materials.

## Figures and Tables

**Table 1:** Physical and chemical properties of lignite sample tested.

Parameter, Unit	Value
**Proximate analysis**	-
Total moisture ^1^, % *w*/*w*	12.97
Ash ^1^, % *w*/*w*	23.07
Volatiles ^1^, % *w*/*w*	37.53
Fixed carbon ^2^, % *w*/*w*	26.43
**Ultimate analysis**	-
Sulfur ^3^, % *w*/*w*	2.84
Carbon ^4^, % *w*/*w*	42.63
Hydrogen ^4^, % *w*/*w*	3.21
Nitrogen ^4^, % *w*/*w*	0.34
Oxygen ^2^, % *w*/*w*	15.37
**Heating value**	-
Higher heating value ^5^, kJ/kg	16,602
Lower heating value ^5^, kJ/kg	15,585

^1^ PN-G-04560:1998 with the use of automatic thermogravimetric analyzers LECO (St. Joseph, MI, USA): TGA 701 and MAC 500; ^2^ PN-G-04516:1998 calculated by difference; ^3^ PN-G-0484:2001 with the use of an automatic analyzer TruSpec S by LECO; ^4^ PN-G-04571:1998 with the use of an automatic analyzer TruSpec CHN by LECO; ^5^ PN-G-04513:1981 with the use of LECO calorimeters: AC-600 and AC-350.

**Table 2:** Properties of porous structure of lignite chars determined with the use of nitrogen sorption isotherm at −196 °C and carbon dioxide sorption isotherm at 0 °C.

Sample No.	Pressure under CO_2_ Atmosphere, MPa	Multi-Point BET, m^2^/g	Average Pore Diameter, nm	Total Pore Volume, cm^3^/g	MC Volume, cm^3^/g	MC Area, m^2^/g
1	0.1	197	7.46	0.368	0.058	177
2	1	215	6.81	0.366	0.065	206
3	2	227	6.41	0.365	0.066	207
4	3	244	6.57	0.400	0.068	212
